# Implant-supported fixed prostheses in a Patient with Osteogenesis Imperfecta: A 4-year follow-up

**DOI:** 10.4317/jced.53958

**Published:** 2017-12-01

**Authors:** Mario Caicedo-Rubio, Elvira Ferrés-Amat, Eduard Ferrés-Padró

**Affiliations:** 1 DDS. Diplomate in Implantology and Oral Rehabilitation. Director of Clínica Dental Naturdent. Ansoain, Navarra. Spain; 2 DDS, PhD. Service of Oral and Maxillofacial Surgery. Hospital de Nens de Barcelona. Barcelona. Spain. Department of Oral and Maxillofacial Surgery, Faculty of Dentistry, Universitat Internacional de Catalunya. Barcelona, Spain. Institut Ferrés Amat. Barcelona, Spain; 3 MD, DMD, OMS, PhD. Head of the Service of Oral and Maxillofacial Surgery. Hospital de Nens de Barcelona. Barcelona. Spain. Director of Institut Ferrés Amat. Barcelona, Spain

## Abstract

Osteogenesis Imperfecta (OI) is a rare autosomal dominant connective tissue disorder in wich, the bone quality and density is affected. OI includes some metabolic disorders and have a wide range of clinical presentations. In Osteogenesis Imperfecta bone has a very low density and it is a disorder currently treated with bisphosphonates. Quality and quantity of bone is important for establishment of osseointegration in dental implants. There are few reported cases in the literature. This is a case report of a 61 year-old man with grade IV OI, rehabilitated with implant-supported fixed prostheses in the posterior right and left mandible, whithout bone grafts. At the 4-year follow-up, clinical and imaging study showed no evidence of pathology in the peri-implant tissues. The final outcome is a correct occlusion and masticatory function. This case shows that dental implants may be a treatment option in this patients, however there is still quite limited scientific evidence.

** Key words:**Osteogenesis imperfecta, osteoporotic bone, dental implants, bone fragility, bisphosphonates, drilling technique.

## Introduction

Osteogenesis Imperfecta (OI) is a connective tissue hereditary disorder which comprises a broad spectrum of phenotypic presentations. It is a heterogeneous genetic disorder which affects mainly type 1 collagen (1). OI clinical management is multidisciplinary and it reaches from physical rehabilitation and surgical procedures, audition management, dental and lung disorder as well as the use of bisphosphonates (BP).

Glorieux *et al.* in 1998, suggested using the BP therapy for OI patients. Their treatment was very limited to small groups of patients (2). Although different clinical uses of BP have been reported since 1990 (3), it is in 1995 when the first case of the fail of osseointegration in implant surgery in a patient undergoing BP therapy for Osteoporosis treatment was published (4). It was in 2001 when secondary effects of BP were mostly reported, as at this moment their use became very popular and osseous disorders were diagnosed in patients undergoing this kind of treatment (3-5), already in 2003 definitive diagnosis of Maxillary Osseous Necrosis related to BP was established (4), because of this, identifying patients under risk has become very important for health professionals, pharmacy industry and health regulating organizations to avoid the development of mentioned complications (4,5).

Most of the affected patients were the ones who underwent some kind or invasive dental treatment and under intravenous (IV) BP therapy, as zoledronic acid and pamidronate. In 2006 Maxillary Osseous Necrosis was reported secondary to the use of oral bisphosphonates as Alendronate (4).

Recent data indicate a prevalence of Maxillary Osteonecrosis Induced by Bisphosphonates (MOIB) of 1 to 5% with intravenous bisphosphonates therapy, and from 0.001 to 0.01% with oral bisphosphonates, as well as from 0.09 to 0.34% after an invasive dental treatment (3). It has been found that the most affected structures are the mandible in a 78%, the maxilla 16% and both can be affected in a 5%; in 52% it occurred in patients who underwent a dental extraction and in 48% spontaneously (3-6).

It is known that treatment with intravenous via BP will enhance toxicity and susceptibility to develop a MOIB, on the other hand, when medication is administered orally the risk will be related to the ingested dose and the exposition time. There will be more risk as the duration of the treatment is longer; each decade of treatment will increase in a 9% the risk for the patient (5).

Patients receiving BP intravenous therapy and also undergoing some kind of maxillary surgery, have 7 times more risk to develop MOIB than those who do not undergo any procedure (5). The uncertainty in their treatment which the professional must face is due to the possible mandibular osteonecrosis, to the difficulty in soft tissues cicatrization and the potential lack of implant osseointegration due to the low density of bone in this type of patients.

Long term results are unpredictable (7). We have not found in the literature any protocol to prepare the implant site in patients with Osteogenesis Imperfecta. The aim of this work is to describe the protocol used to insert implants in a patient with OI treated with intravenous bisphosphonates, the rehabilitation with partial implant-supported prosthetics and its favorable evolution-without complications- in 4 years.

## Case Report

**Figure 1:**
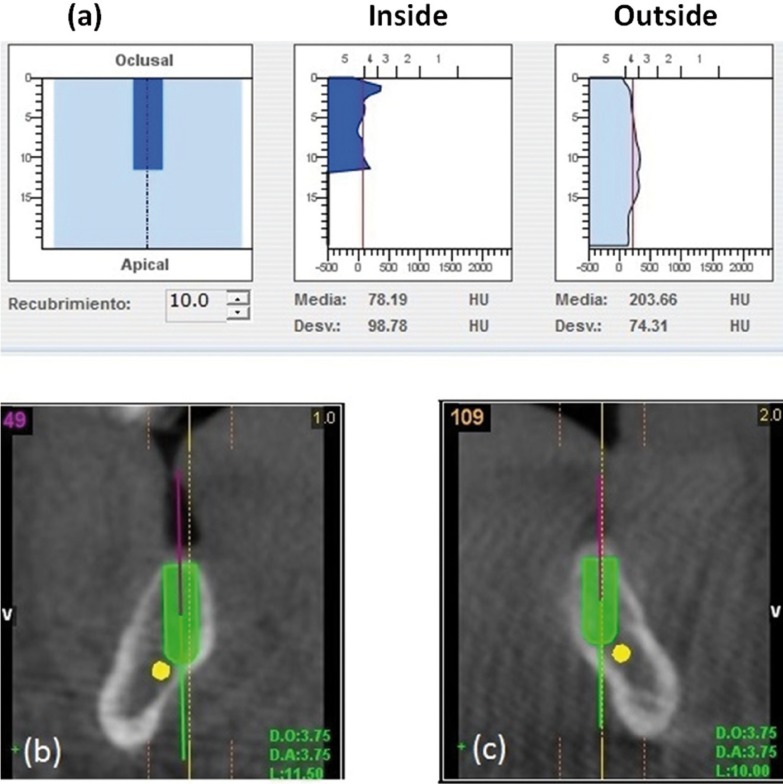
CBCT (a) Bone density in cancellous bone. (b) Planning implant 46. Bicortical anchorage. (c) Planning implant 36. Bicortical anchorage.

The case involves a 61 year old, male patient, who arrived in the dental clinic because he was not able to chew properly due to his removable prosthesis. The patient, with grade IV Osteogenesis Imperfecta, desired to have a better situation possibly replacing his removable prostheses for a fixed option. The patient is smoker, 20 cigarettes a day, with previous stroke/cerebrovascular accident (CVA) in 2007, head of the femur prostheses in 2009, and multiple fractures in his legs. His OI was treated with Aclasta® 5 mg. (zoledronic acid) in solution for intravenous perfusion every six months since 2011. Last dose in June 2015, his physician suspended the treatment. Adiro® 300 mg. (acetylsalicylic acid) tablets every 24 hours since 2007 until nowadays and Hidroferol® vials 1.5 ml (calcifediol) every 15 days since 2009 until nowadays. Intraoral clinical exam: He presents partial edentulism, resi-dual dental roots in third quadrant, cervical caries in 16 with severe mobility, poor oral hygiene, upper removable partial prost-hesis. Radiographic Exam: Orthopantomography (OPG): root fragments in 36 area, caries with pulp involvement in 16, cervical caries in 13. Dental absence in the anterior area of the second quadrant and molar area of first, second, third and fourth quadrant. Cone Beam Computed Tomography (CBCT) : Very low density bone is observed in the upper maxilla, 158 Hounsfield Units (HU) osseous density in cancellous bone, 670 HU in cortical bone. And in the lower jaw, 78 HU osseous density in cancellous bone, 443 HU in cortical bone (Fig. 1).

After evaluating the risks, we decide with the patient, to accomplish the following treatment plan: Oral hygiene instructions, elimination of local factors, tobacco, bacterial plaque and caries, root fragments of 36 extraction, extraction of 16, partial fixed prosthesis in the upper jaw with metal-ceramic crowns with attachments in 13-12-11-21, Removable Partial upper posterior prosthesis with attachments, Partial Fixed Lower Prosthesis with metal-ceramic crowns from 34 to 45, Implant supported prosthesis on implants C1 (sand-blasted and acid-echted surface, conical shaped, conical connection and platform switching) of MIS Implants Technologies LTD, Shlomi, Israel, two implants area 36-35 (MIS C1 3.75x10), one in 46 (MIS C1 3.75 X 11.5), MIS Multiunit abutments in third quadrant, MIS transepithelial abutment. Design for cement-screwed crown in 46, Crowns on implants 36-35 and 46. The patient signed two specific informed consent, one for the dental extractions and the second one for the implant insertion.

Objective of the treatment: Functional reestablishment of the patient dentition by an oral rehabilitation formed by a fixed implant-supported prosthesis in the lower molar area and dental-supported in the upper.

Dental extractions: Were performed under antibiotic prophylaxis of amoxicillin 500 mg. capsules every 8 hours since one day before dental extraction and until 6 days after. In October 2011were extracted the radicular rests of the left mandibular first molar-36 (four month after the last BP dose)and in april2012 was extracted the right maxillary first molar-16 (four month after the last BP dose).

Description of the technique of low density bone drilling in OI.

Treatment is started 2 months after the last dose of bisphosphonates, with an antibiotic prophylaxis of amoxicillin 500 mg. capsules every 8 hours since one day before implant placement and until 6 days after. The adjacent mucosa was dried followed by application of topical anesthetic gel (benzocaine 2%) with the help of cotton applicator for about 30 s. Later, 1.8 ml of anesthetic solution (2% lidocaine with epinefrine 1:50,000 buccally [0.9 ml] and lingually [0.9 ml], was injected under aseptic conditions using a European thread M6x0,75 (0,3x25mm) gauge needle at a rate of approximately 1 ml/min. To achieve effective buccal and lingual anesthesia, the procedure was delayed for at least 5 min. The plan is to get a bicortical anchorage, the implant will not act as a compactor, a protocol of drilling with the complete sequence and new drills at low speed is followed (120 RPM), plenty of irrigation with sterile physiological saline solution, Three C1 implants were placed subcrestal (submerged), taking place in two surgical times, leaving 6 months between these two phases to allow osseointegration.

Primary anchorage in the lingual cortical and in the cortical apical to the implant (mylohyoid line) was aimed at the moment of the implant placement. The cancellous bone presents very low density.

**Figure 2:**
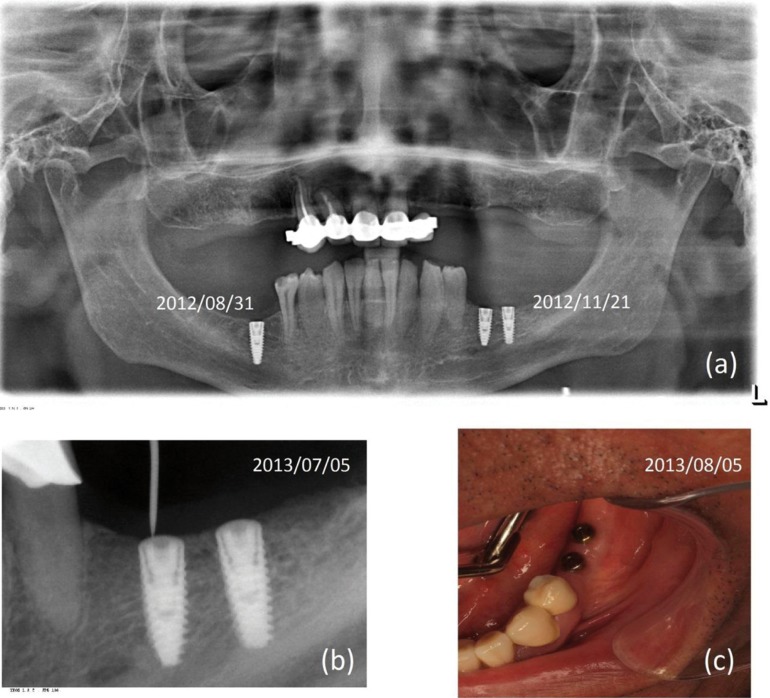
Radiographic and soft-tissue controls. (a) OPG with placement dates of implants. (b) Integrated bone implants are seen. Exploration for the placement of healing abutments. (c) Healthy soft tissues with healing abutments.

Implants at third and fourth quadrant were placed in different times. The aim was to check if the healing process was favorable, due to the risk of osteonecrosis. MIS C1 Implant on area 46 of 3.75x11.5 and insertion torque of 50 Ncm was placed in August 2012 (two month after the last BP dose). Once the correct healing of the soft tissues was checked and after observing the osseointegration on the x-rays, two MIS C1 implants of 3.75x10 with a programmed torque of 50 Ncm, were placed on area 36 and 37 in November 2012 (five month after the last BP dose), and one month after the implant insertion the zolendronic acid treatment was restarted by his physician. Prosthetic Rehabilitation: Placement of healing abutments, checking implant stability, taking impressions, trying on the structure, trying the ceramic, occlusion adjustment, placement of crowns on implants in September 2013 with a torque of 35 Ncm, control after a month (8-10) (Fig. 2).

Recommended Maintenance: Oral hygiene instructions were explained. Daily use of a water jet. Daily use of a mouth rinsing solution of Chlorhexidine Digluconate 0.12%. Soft diet. Monitoring and professional dental cleaning every six months.

**Figure 3:**
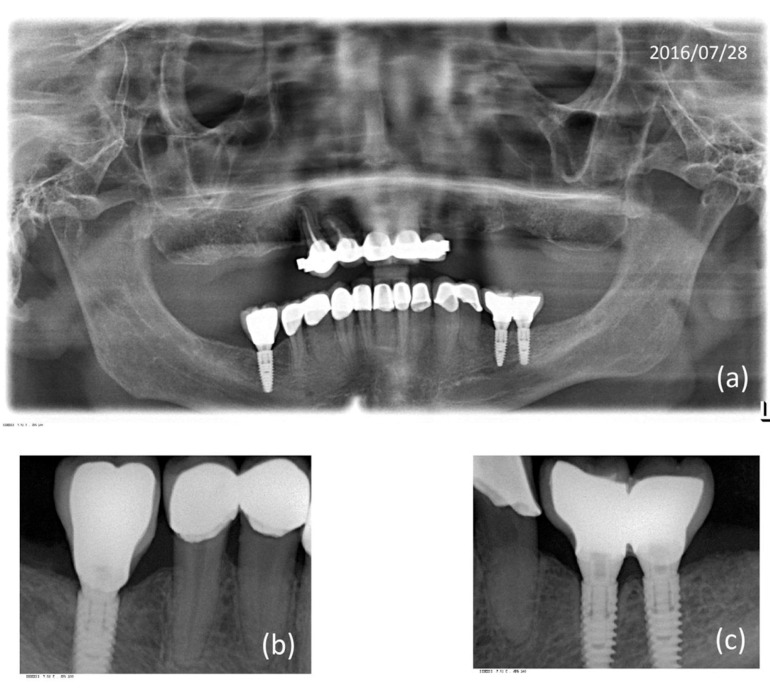
Radiographic control dated July 28, 2016. (a) OPG with fixed prosthesis (b) Rehabilitation implant zone 46. (c) 36-37 Implants zone. 1.25 mm of bone loss is observed in the implant 36.

Clinical and radiological examinations were performed in order to follow up the case. Stability of the peri-implant tissues is found at the follow-up visits, the patient is very happy with the results, he refers to have a very diverse diet and keeps on smoking. The existing keratinized gum is stable, although insufficient at implant on area of 36. Oral hygiene is deficient. On January 2014 follow up, a slight bone loss at the crestal area of implant on 36 area starts to be noticeable, generalized gingival inflammation is observed, in every teeh, related to the presence of bacterial plaque, it is observed that peri-implant tissues are in good health. In later follow-ups until July 2016 peri-implant tissues are stable, with a loss of 1.25mm of crestal bone on the area of implant 36, the same level that was measured in 2014 (Fig. 3).

## Discussion

Treatment with dental implants in patients with OI is extremely rare. We have been able to find only six cases previously published in the scientific literature (7,8,11-14). The intravenous (IV) administration of bisphosphonates implies a special situation, which relevance is associated to the time of exposure of the patient to this drug therapy, under or over 3 years and if he is exposed to OMIB associated risk factors, smoking, bad oral hygiene, periodontal disease (5).

Implant insertion in maxillary bones on patients undergoing OI and who are treated with IV bisphosphonates is considered by some authors an unfeasible treatment (15). However we need to know the dose of IV BF, the zolendronic acid used in the treatment of the patient in OI is 5 mg every 6 months (Aclasta®), and in patients with bone-metastases is 4 mg every 3 to 4 weeks. This difference of dose and dosage regimen can explain the risks and benefits of continuing bisphosphonate therapy and justify the treatment decision made individually with the patient (5).

With these reported cases, a great possibility opens for future patients with this pathology, they will be able to be rehabilitated with fixed prosthesis on implants although it is necessary to develop more studies and to scientifically check its feasibility.

It is very important that both dentists as every healthcare professional can manage the basic principles for preventing osteonecrosis caused by bisphosphonates, by the acomplishment of precise medical records and establishing a multidisciplinary management in patients susceptible to develop this illness, in such a way that wrong, misinterpreted or late diagnosis and, at the end, failed treatments that could worsen the course of this condition will be avoided (15).

## Conclusions

This case shows that dental implants may be a treatment option in patients with osteogenesis imperfecta, however there is still a limited scientific evidence and special attention must be focused to the potential risks of the intravenous bisphosphonates therapy and the patient must know that it is an individual case.
